# *Listeria monocytogenes*: A Foodborne Pathogen with Implications for One Health and the Brazilian Context

**DOI:** 10.3390/microorganisms13102280

**Published:** 2025-09-30

**Authors:** Felipe Gaia de Sousa, Rosely Maria Luzia Fraga, Ana Cristina Ribeiro Mendes, Rogério Carvalho Souza, Suzane Lilian Beier

**Affiliations:** 1 Department of Veterinary Clinic and Surgery, Veterinary School, Federal University of Minas Gerais—UFMG, Belo Horizonte 31620-295, Brazil; fgaias@ufmg.br; 2 Department of Veterinary Medicine, Faculty of Veterinary Medicine, Pontifical Catholic University of Minas Gerais—PUC Minas, Belo Horizonte 30140-002, Brazil; fraga.rosely@gmail.com (R.M.L.F.); anacrmen@pucminas.br (A.C.R.M.); rogeriocs@pucminas.br (R.C.S.)

**Keywords:** bacteria, biofilms, foodborne diseases, food safety, good manufacturing practices, listeriosis

## Abstract

Foodborne diseases (FBDs) represent significant public health concerns as they are conditions associated with deficient manufacturing practices. They comprise important diseases with acute or chronic courses, frequently occurring in outbreak form and associated with significant gastrointestinal disorders. FBDs are related to infrastructure and organizational issues in urban centers, such that contamination in food processing facilities, lack of access to basic sanitation, and social and financial vulnerability are some of the factors that favor their occurrence and the demand for health services. Among the agents associated with FBDs is *Listeria* sp., especially *Listeria monocytogenes* (*L. monocytogenes*). The objective of this article is to characterize *L. monocytogenes* and its potential impact on One Health, given its importance as a significant foodborne pathogen. A thorough scientific literature search was conducted to obtain information on the subject, aiming to assist in the verification and presentation of evidence. *L. monocytogenes* is a pathogen with specific characteristics that ensure its adhesion, adaptation, growth, and survival on various surfaces, such as biofilm formation ability and thermotolerance. Several diagnostic methods are available for detection of the agent, including enrichment media, molecular techniques, and subtyping evaluation. Its control represents a significant challenge, with critical implications due to bacterial perpetuation characteristics and the implementation/monitoring of sanitization programs and commercialization of animal-derived products (POAO). Thus, vulnerable and susceptible populations are more exposed to foodborne pathogens due to health-related determinants, such as inadequate sanitation, poor food safety control, and insufficient personal hygiene. The pathogen’s persistence and difficulty of control represent a significant public One Health threat.

## 1. Introduction

The consumption of products derived from animals (POAO), such as meat, milk, and dairy products, drives the global economy, with significant increases in production and trade expected. Consequently, the final consumer values the quality of marketed products and may express concern regarding both the industrial production stages and the subsequent processes of storage and commercialization. Depending on changes in the processing stages of POAO—from slaughter to inspection, packaging, and cooking, among others—these products are susceptible to both internal and external contamination, compromises the attainment of a high-quality final product, directly impacting both sales and consumer health. Foodborne illnesses, also known as foodborne diseases (FBDs), are characterized as a group of diseases sharing the common factor of transmission through contaminated food. FBDs are frequently associated with systemic health, tending to occur in malnourished or weak patients or those with immune system deficiencies, making them more prone to clinical effects [1].

FBDs can have various causes such as biological, chemical, or physical agents and may present acute or chronic courses [2]. From a biological perspective, the agents most commonly involved are bacteria, viruses, and parasites such as protozoa [2]. FBDs can be classified as acute conditions—where the incubation period is short, lasting hours to months, usually resulting from a single exposure—or chronic conditions resulting from multiple exposures [2]. Acute cases generally cause a range of clinical symptoms, mainly related to the gastrointestinal system such as vomiting and diarrhea, as well as more nonspecific symptoms like lethargy and dehydration [2]. It is important to note that persistent cases may be associated with structural issues in urban centers, including poor infrastructure such as inadequate sanitation, vulnerability situations, limited access to health programs, and low quality of life, among other factors. The persistence of FBDs in these cases is specifically related to prolonged contact with the transmitting agents, resulting in a long-lasting and exacerbated process. Thus, FBD mortality rates may be directly related to the population’s location and urban infrastructure.

FBDs can occur in the form of outbreaks, representing rapidly spreading and concerning situations that overload healthcare services. Additionally, due to insufficient hygiene steps—particularly individual hygiene—the disease may spread further. The presence of outbreaks allows better characterization and understanding of the disease, making it necessary to establish causal factors including biological, temporal, and local aspects. However, this requires complete and accurate information. More than 200 diseases can be transmitted through food, which emphasizes the need for greater attention to food preparation and hygiene [3]. The WHO [4] estimates that approximately 600 million people are affected by FBDs annually, with an estimated 420,000 deaths; for children, the death toll is estimated at around 125,000 per year. Food insecurity is a serious problem that threatens the quality of life of countless individuals, often being associated with reduced food safety due to fluctuations in the availability of quality food, which places them at high risk. In this context, good production practices, from processing to storage for commercialization, are essential to ensuring safe and healthy food consumption. Especially concerning POAO, *Listeria monocytogenes* stands out as an important foodborne pathogen, along with *Escherichia coli* and its strains.

*Listeria* spp. is characterized as a group of bacteria found in various environments such as soil, vegetation, and water bodies, Gram-positive, with some species described as pathogenic, including *L. monocytogenes* [3,5,6]. *L. monocytogenes* is associated with infections in humans and animals, considered a significant pathogen impacting both global public health and the economy [5]. It shows high morbidity and mortality rates [7,8,9]. *Listeria* spp. are important foodborne pathogens due to their association with sporadic cases, outbreaks, and mass food product recalls [9,10,11]. Safe disposal of contaminated products is a critical step to prevent the spread of bacterial agents; however, it represents a substantial economic loss for producers and vendors due to the inability to consume the food and recoup costs. One of the concerns with *L. monocytogenes* and other bacterial agents causing FBDs lies is their ability to form biofilms, which are communities of bacteria adhered to different surfaces. These biofilms can act as sources that spread harmful agents due to inadequate sanitation [12,13]. Therefore, the repeated use and handling of food in areas prone to biofilm formation without proper cleaning is an important source of infection. For Hua and Zhu [14], cross-contamination can occur on surfaces and in food preparation environments, highlighting the need for proper sanitation practices. Concerns about *L. monocytogenes* also relate to its resistance to disinfectants and/or antimicrobials used during sanitation processes [15,16,17,18,19]. This article aims to characterize *L. monocytogenes*, including its impact as a foodborne pathogen associated with FBDs in the context of public health.

## 2. Listeria and Pathogenic Strains

The presence of *Listeria* spp. in food processing environments poses a threat to human health, especially due to its harmful potential, survival capabilities, and pathogenicity [20]. Food safety depends on the application of good manufacturing practices, from raw material to commercialization [21]. The genus Listeria belongs to the family Listeriaceae, is monophyletic, and can be subdivided into *sensu stricto* and *sensu lato* groups [6]. Manyi-Loh and Lues [15] state that “as an overview of *Listeria* species (*L. monocytogenes*), the genus Listeria comprises 20 species, which can be categorized further into non-pathogenic species, (including *L. thallandensis*, *L. costaricensis*, *L. goaensis*, *L. newyorkensis*, *L. booriae*, *L. riparia*, *L. grandensis*, *L. floridensis*, *L. cornellensis*, *L. aquatica*, *L. weihenstephanensis*, *L. fleischmannii*, *L. marthii*, *L. rocourtiae*, *L. ivanoii*, *L. seeligeri*, *L. innocua*, *L. grayi*, and *L. welshimeri*), and the main pathogenic species, *L. monoctyogenes*”. For Oliveira et al. [22], two species are considered pathogenic to humans: *L. monocytogenes* and *L. innocua*. Regarding *L. monocytogenes*, its growth is favored under optimal conditions such as temperature (0.4–45 °C), low water activity, and a pH range between 4.4 and 9.6 [3,23,24]. 

Ravindhiran et al. [3] state, “*L. monocytogenes* is a small Gram-positive rod, facultative anaerobe, invasive, non-spore-forming, and intracellular foodborne pathogen known to cause a systemic disease called listeriosis in humans and ruminants. They are motile at 24 °C to 28 °C due to peritrichous flagella and are non-motile above the temperature range of 30 °C.” The thermotolerance of *L. monocytogenes* is a key factor favoring its development due to its ability to survive and grow across a wide temperature range [25,26]. The isolation of the bacterium from refrigerated food products confirms the pathogen’s ability to survive at low temperatures, such as −1.5 °C, characterizing it as psychrotrophic [5,15,27]. Evidence indicates a certain complexity regarding the causal factors that allow *L. monocytogenes* to persist even at low temperatures, but it is suggested that this may be related to substances with cryoprotective properties, cold shock proteins, and cellular modifications [28]. The pathogen behaves differently under low-temperature conditions, adapting to the new environmental constraints [29]. More detailed information about the pathogen’s cold tolerance can be found in the review by Liu et al. [30].

Acidity level is another factor that disfavors *L. monocytogenes*, since the use of organic acids is a common practice in food preservation [15,31]. The application of acidulants provides microbial control by increasing the abundance of acidic anions that inhibit the growth of pathogens [32]. The use of acids raises the concentration of hydrogen protons, which impairs bacterial cell growth and development [15]. These bacteria often exhibit specific responses to acidity, adapting to the established condition and developing survival mechanisms [15]. This adaptation is linked to their ability to grow and persist, as the biological protonation process caused by increased proton concentration hinders bacterial function and perpetuation [33]. Thus, bacteria can be classified as acid tolerant or acid resistant, with variability in the ideal pH range for survival [33]. *L. monocytogenes* is particularly notable for its ability to adapt to acidic environments through metabolic and homeostatic processes that regulate bacterial intracellular pH, enabling growth and development [33]. These conditions allow the bacterium to adapt, grow, and persist on surfaces, especially those associated with animal-derived products, making its elimination under extreme conditions challenging [34].

*Listeria* spp. present morphological similarities, although biochemical tests allow for their differentiation [3]. For Orsi et al. [6], *Listeria* spp. *sensu stricto* are “gram-positive short rods, catalase positive, showing a positive result for the Voges–Proskauer test (which assesses the production of acetoin from glucose fermentation), oxidase negative, unable to reduce nitrite or nitrate, able to ferment D-arabitol, methyl-α-D-glucopyranoside, D-maltose, D-lactose, unable to ferment inositol, D-mannitol, and L-arabinose.” Orsi et al. [6] also highlight that *L. monocytogenes* exhibits β-hemolytic activity and phosphatidylinositol-specific phospholipase C (PI-PLC) activity, the former associated with hematological destruction and the latter related to cellular function. Furthermore, Karthikeyan et al. [35] note that the survival and cellular replication abilities of *L. monocytogenes* contribute to its dissemination and pathogenicity, as well as to challenges in control measures. Ravindhiran et al. [3] report that the disease can present in invasive and non-invasive forms, with the former affecting immunocompromised individuals and the latter healthy individuals. *L. monocytogenes* is an intracellular bacterium found in various environments, present in animal-derived products, vegetation, and highly decomposed matter [36]. There is evidence of the bacterium in “soil, water, vegetation, sewage, animal feeds, farms, and food-processing settings” [6]. The authors emphasize that bacterial dissemination is influenced by the environmental characteristics of each habitat. Hafner et al. [37] demonstrate that pathogenic strains such as *L. monocytogenes* travel between hosts through different environments, aiding their bioprogression, perpetuation, and pathogenic efficiency. Its lifestyle can be classified as saprophytic or host-dependent [34], meaning its survival relies on parasitism. Manyi-Loh and Lues [15] also state that the agent has the ability to survive for extended periods in food processing environments, making it as an important pathogen that is difficult to control.

### Sources of Contamination and Clinical Presentations in Humans and Animals

Considered an FBD of occasional occurrence, the illness mostly presents in the form of outbreaks following contact with the pathogenic agent, particularly through consumption. In cases of listeriosis arise from the ingestion of food products such as meat, milk and its derivatives, as well as seafood [5,38]. It is also noteworthy that some evidence has pointed to transmission via vegetables and fruits in household settings [39,40,41]. The study by Tsitsos et al. [18] demonstrated that pathogen spread can occur through water used in the cleaning of carcasses and hides. Quereda et al. [5] state that “foods mostly associated with foodborne listeriosis include RTE products that: (i) support growth of *L. monocytogenes*; (ii) are consumed without receiving any antibacterial treatment (e.g., thermic treatment); and (iii) have long refrigerated shelf-life.” Thus, the occurrence and development of the foodborne pathogen is plausible. The article published by the same author provides a more detailed demonstration of sources of *L. monocytogenes* contamination, including evidence from reports of ingestion of contaminated food product and derivatives, as well as isolation sites such as soil, farms, and insects. Consequently, the extensive ability of *L. monocytogenes* to adapt and survive in different reservoirs and fomites becomes more evident, making it difficult to control even under adverse conditions (acidity and temperature) [22]. Listeriosis in humans, as previously mentioned, is considered an important disease due to its capacity to cause organic damage through intestinal alterations and consequences such as dehydration and lethargy. Moreover, although it is not a saprophyte of the oral cavity, it can be found there if hygiene practices are poor [22]. It is highlighted that these bacteria can develop in the oral cavity after prior contact, using it as a nutrient substrate for growth and adhesion; thus, saliva functions as a protective agent against bacterial colonization [22,42,43]. Quereda et al. [5] state that the symptomatology of *L. monocytogenes* also depends on pathogen characteristics such as strain pathogenicity, which explains the differing clinical presentations in severity. In addition to strain pathogenicity, factors such as the ingested dose of the pathogen, genetic variability, systemic and immunological health status, or any food properties that modify the pathogen or host, are also relevant [44].

To date, health surveillance data have shown variability in establishing the minimum contamination levels of *Listeria monocytogenes* across different types of food, representing a significant gap in the detailed understanding of foodborne listeriosis [6]. Regarding listeriosis, the dose–response relationship of the disease remains poorly characterized; however, in the study by Pouillot et al. [45], the authors highlight that “considering classes of virulence leads to estimated relative risks (RRs) of listeriosis following the ingestion of 1000 bacteria of “less virulent” vs. “more virulent” strains ranging from 21.6 to 24.1, depending on the sub-group”. Underreporting of information related to FBDs is a significant obstacle due to incomplete outbreak investigations. However, evidence points to infectious doses of 10^7^–10^9^ colony-forming units (CFUs) in healthy patients and 10^5^–10^7^ CFUs in vulnerable individuals [46,47,48,49,50]. Based on these data, it is reasonable to say that healthy individuals require higher *L. monocytogenes* CFU concentrations to become infected compared to immunocompromised individuals. This may explain why individuals who consume contaminated food do not develop clinical symptoms, particularly when ingested CFU loads are low and there are no factors favoring bacterial growth [50]. The study by Grif et al. [51] already showed that fecal transmission of *L. monocytogenes* in humans does not correlate with evident clinical disease. The recent study by Hafner et al. [37] confirms that fecal shedding of *L. monocytogenes* is not associated with clinical symptoms, demonstrating that in a cohort of nearly 1000 asymptomatic healthy donors, the pathogen was detected in 10% of samples. The authors emphasize that fecal occurrence of *L. monocytogenes* is frequent and dependent on the intestinal microbiome, representing a condition worthy of attention due to its association with virulence markers, especially in pathogenic species.

The ability of *L. monocytogenes* to disseminate and survive in different environments enables it to be a pathogen likely found during food processing and manufacturing stages [5,20]. The bacterium can be found in “natural environments, farms, soil, water, silage, decaying vegetables, human and animal feces and tissues, food processing industries, and processed food product” [5] which is strongly associated with various transmission routes, as it can be spread across diverse surfaces and hosts [3]. Velge et al. [52] describe that the pathogen’s incidence increases in relation to human–animal interactions. Evidence already points to a high genetic diversity of *Listeria* spp. in fomites and rural production animals [53,54], so it is expected that these pathogens adapt to the conditions of various environments and hosts. Various animals can be contaminated with the pathogen, including goats, sheep, canines, and wildlife, among others [3]. It is important to emphasize that, as described by several authors, production animals may present the disease asymptomatically, thereby contributing to pathogen dispersal via feces, which can contaminate meat products, dairy and derivatives, as well as equipment and other areas [55,56,57]. Hafner et al. [37] suggest that the ability of hosts to remain asymptomatic may be directly related to pathogen transmission, implying that the agent possesses genetic selection ability considering the host–pathogen axis. Furthermore, Quereda et al. [5] highlight that the pathogen can be introduced into POAO establishments as a “result of cross-contamination by workers (human carriers), transportation of animals (fecal shedders), raw food (e.g., milk), and materials from crops, soil, and silage.” The possibility of introduction into POAO processing industries may be related to its ability to persist in environments and survive for long periods, representing an important transmission route [5]. Ferreira et al. [58] point out that the main factors associated with pathogen survival and spread relate to its ability to produce biofilms, resistance to different temperatures, resistance to disinfectants, and presence in hard-to-clean locations and equipment. Ideally, fomites and natural reservoirs of *L. monocytogenes* should be well described to help prevent its introduction into POAO establishments and rural properties [5]. Therefore, it is necessary that such locations be constantly monitored to understand the spatiotemporal and causal distribution of the disease, so that its severity can be contained and its spread reduced or avoided, minimizing the risk of contamination to POAO. 

The clinical presentation of listeriosis in humans is variable and dependent on factors already mentioned, such as immunological status. Additionally, it is important to note that mammals serving as natural hosts of the pathogen may have the capacity to limit bacterial growth or even control the spread of the agent to other sites [5]. D’Orazio [59] emphasizes that the disease presentation can be systemic, especially in patients with impaired immune function who are effectively contaminated with high loads of CFUs. In humans, *Listeria* spp. can cause distinct clinical presentations, including cases of gastroenteritis associated with fever, septicemia, maternal–fetal infections, neurological symptoms, and localized infections [5]. From a gastrointestinal perspective, *L. monocytogenes* induces acute, self-limiting diarrheal episodes, with most outbreaks associated with loads exceeding 10^9^ CFUs/mL, an average incubation period of one day, and an attack rate of up to 72%. It is noteworthy that gastrointestinal-related cases typically present with fever, gastroenteritis, and joint and muscle pain, as well as headache [60,61,62,63,64,65,66,67]. Septicemia and maternal–fetal axis infections are closely associated with *L. monocytogenes*’ ability to penetrate key tissue barriers, such as the blood–brain, placental, and intestinal barriers [5]. The MONALISA study [68] confirmed these clinical findings, showing that among the 818 cases evaluated, most were related to septicemia, while the remaining were associated with neurolisteriosis and maternal–fetal infections. The average incubation period for invasive *L. monocytogenes* infections varies depending on the clinical presentation: approximately one week on average, with variation as follows—septicemia: 2 days (range: 1–14), neurolisteriosis: 9 days (range: 1–14), and maternal–fetal infections: 27.5 days (range: 17–67) [64]. Septicemia caused by *L. monocytogenes* presents similarly to other bacterial bloodstream infections, characterized by nonspecific signs such as fever, vomiting, and lethargy [5]. Neurological involvement typically includes rhombencephalitis, abscess formation, neck stiffness, movement disorders such as ataxia, and seizures [5].

Clinical manifestations of *L. monocytogenes* infection during pregnancy are often nonspecific, resembling flu-like symptoms or even pyelonephritis [5]. The main concern regarding *L. monocytogenes* infection in pregnant women lies in the risk of fetal infection [5,66,69,70,71]. This can occur via transplacental transmission due to systemic or uterine infection, or during delivery through contact with vaginal and/or anal secretions [66,70,71]. Koopmans et al. [72] report that neonatal neurolisteriosis can present as either early-onset (within 48 h after birth) or late-onset (after 48 h). It is important to highlight the potential for fatal outcomes or the development of granulomatosis infantiseptica in these cases [66,70,71]. Newborns may develop neonatal meningitis up to 14 days postpartum if infected during delivery [66,70,71]. Although maternal neurolisteriosis can occur, it is rarely fatal for the mother; however, significant fetal or neonatal complications are commonly observed [68]. Regarding localized infections, Charlier et al. [68] emphasize that these account for only a small number of cases compared to other clinical forms. Quereda et al. [5] note that this presentation is considered an occupational hazard, albeit rare, typically occurring during labor. These infections are characterized by papular or vesicular skin eruptions, sometimes associated with pustules [73,74]. They are self-limiting, localized, mild, and not typically associated with pruritus or pain [73,74]. As stated by Koopmans et al. [72], such infections may be “seen in farmers or veterinarians, contracted by direct exposure to infected lochia, placenta or aborted fetuses from materno-fetal cases of *L. monocytogenes* infection in ruminants”. Several other clinical forms of human listeriosis are also currently described [5].

From the veterinary perspective, most *L. monocytogenes* infections in animals are subclinical, with outbreaks or isolated cases occurring sporadically [5]. Clinical manifestations in ruminants are generally similar to those observed in humans [75]. Rhombencephalitis is the most common form in ruminants, while septicemic forms are less frequent and typically limited to neonates [5]. In these cases, the pathogen ascends to the brainstem via the trigeminal nerve, leading to cranial nerve paralysis, lethargy, and the so-called “circling disease”. Inflammation of the meninges and/or the brain may also occur, as reported by Oevermann et al. [76] and Henke et al. [77]. Quereda et al. [5] also note that “abortion is usually late term, and although less frequently reported, mastitis has been associated with *L. monocytogenes* infection”. In sheep, gastrointestinal disturbances may also occur [78]. Laven and Lawrence [79] have reported ocular disorders in cattle following exposure to contaminated feed. In swine, the most commonly observed presentations are septicemic forms [75,80].

## 3. Pathogenesis of Listeria Monocytogenes and Dissemination Pathways

### 3.1. Pathogenicity Mechanisms and Virulence Markers

For *L. monocytogenes* to interact with cellular components after entering the host organism, it must rely on a coordinated interaction of virulence factors that enable its survival and persistence [22,81,82]. The pathogen requires a communication pathway that allows it to invade, evade phagosomal destruction, replicate intracellularly, and move within the cytosol, as well as disseminate to neighboring cells [22,81]. Ravindhiran et al. [3] state that “*L. monocytogenes* has followed three different mechanisms to exert its pathogenesis in the human host: (a) an intracellular mechanism involving actin-based motility for cytoplasmic movement and cell–cell spreading; (b) an extracellular mechanism involving a free-living, flagellum-propelled bacterium in the environment; and (c) biofilm formation”. The lifestyle shift of *L. monocytogenes* from saprophytic to infectious involves changes in gene expression that enhance its intracellular pathogenic capacity [22]. Multiple studies highlight the role of surface proteins as key virulence factors, including internalins InlA and InlB, *Listeria* adhesion protein (LAP), and InlP, which facilitate bacterial attachment and entry into nonprofessional phagocytic cells—those that do not perform phagocytosis as a primary function [83,84,85]. Once internalized, the pathogen can escape from the phagosome through the action of vacuolar escape factors such as the pore-forming cytolysin listeriolysin O (LLO), phosphatidylinositol-specific phospholipase C (PI-PLC) encoded by *plcA*, and the broad-spectrum phospholipase C (PC-PLC) encoded by *plcB* [3,86]. The bacterium must also adapt its metabolism, shifting to utilize available substrates such as amino acids, sugars, and peptides [87]. Upon reaching the cytosol, *L. monocytogenes* undergoes robust replication using glucose from the host cell as a primary carbon source [72]. The remarkable review by Meireles et al. [82] offers a thorough and comprehensive insight into the molecular signaling pathways involved in the virulence mechanisms of *L. monocytogenes*.

Through actin cytoskeleton subversion, the pathogen promotes its dissemination using the bacterial surface actin assembly-inducing protein (ActA). Additionally, InlC assists by reducing cellular tension, which facilitates the formation and elongation of membrane protrusions [88,89]. Koopmans et al. [72] describe the presence of “listeriopods”—structures resembling pseudopods—that enable the bacterium to move within the cytosol and spread to adjacent cells. These authors emphasize that *L. monocytogenes*’ ability to disseminate intracellularly serves as a protective mechanism, allowing it to evade extracellular immune responses and modulate the host’s immune defense and infection control. The escape from the phagosome depends primarily on LLO, PI-PLC, and PC-PLC [90]. The release of these virulence factors enables the bacterium to adapt, survive, and persist within the host environment [90]. Among the regulatory elements, PrfA is the most critical transcriptional regulator, orchestrating the expression of virulence genes in response to environmental cues to ensure appropriate regulation of the pathogenic process [22,81]. PrfA activation occurs after the bacterium enters the host cell cytosol, triggering gene expression that supports intracellular replication and spread to neighboring cells [81]. Furthermore, the authors note that “the activity of PrfA is itself carefully regulated by a variety of mechanisms that include transcriptional, post transcriptional, and post translational control”. Overall, Koopmans et al. [72] emphasize that the pathogen’s invasive capacity is largely dependent on surface proteins regulated by the PrfA regulon, with internalins and ActA playing key roles in facilitating invasion and dissemination. More recently, Freeman et al. [91] reported that the pathogen also utilizes phosphotransferase systems to aid in its dissemination and persistence.

Numerous *Listeria* serotypes exist across different lineages, but only serotypes 1/2a, 1/2b, and 4b are associated with human disease [3,6,15,92,93,94,95]. Virulence factors in *L. monocytogenes* may be “scattered across the genome (e.g., the inlA-inlB locus, bsh, inlC, and lap, among others) or clustered in pathogenicity islands LIPI-1, LIPI-3, and LIPI-4” with each island contributing to different clinical outcomes. Furthermore, the bacterium’s adaptive capacity and ability to form bacterial clones (CC) may confer selective advantages depending on the environment [5]. As Quereda et al. [5] highlights, three distinct patterns have been proposed among major *L. monocytogenes* clones: “(i) clones that persist efficiently in food-production environments owing to efficient tolerance to disinfectants and biofilm formation, but with low adaptation to the host (e.g., CC9 and CC121); (ii) clones that are host-associated, exhibiting a low adaptation to food-production environments and rarely harboring disinfectant resistance genes (e.g., CC1 and CC4); and (iii) intermediary clones that may be in the process of transitioning from host-associated to saprophytic lifestyles”. Therefore, the prevalence and behavior of *L. monocytogenes* infections may vary depending on the characteristics of each clone. Furthermore, Pracser et al. [96] highlights that the type of food impacts the survival mechanisms, virulence, and gene expression profile of *L. monocytogenes*. 

#### 3.1.1. Bacterial Stress Response and Virulence Mechanisms

Characterized by its ubiquity, *L. monocytogenes* has the ability to adapt and survive under diverse conditions, particularly those associated with stress [5]. For NicAogáin and O’Byrne [97], its ability to proliferate in various POAO, which undergo stringent preservation and food safety processes—such as high salt concentrations, low pH, refrigeration, and bactericidal light exposure—demonstrates the pathogen’s capacity to continuously develop adaptive mechanisms, including resistance to disinfectant-exposed environments. Furthermore, exposure to acidic pH, high intestinal osmolality, bile, and the microbiota are considered major stress-inducing factors [5]. This remarkable stress resistance is partly attributed to the alternative sigma factor SigB, which plays a crucial role in enabling the bacterium to adapt and survive in hostile environments [98]. One example of its adaptive ability is its tolerance to high-salt environments used for food preservation. The bacterium is capable of growing in media containing up to 3 mol/L of salt, a concentration considered extremely high [5]. It achieves this by accumulating solutes that prevent water loss. Under osmotic stress, *L. monocytogenes* utilizes osmolyte transporters such as glycine betaine *(gbu)* and gene encoding a glycine betaine *(betL)*, which facilitate the accumulation of osmoprotective compounds like glycine betaine [99]. The pathogen’s preference for *gbu* or *betL* is conditioned by the availability of these compounds in the surrounding environment or food matrix [5].

In addition to high salt tolerance, the bacterium is psychrotolerant, meaning it can survive and proliferate at very low temperatures, making refrigeration ineffective in fully inhibiting its growth [5]. As noted by Hingston et al. [100], *L. monocytogenes* is capable of slowing down its metabolism under cold shock while inducing precursors that maintain membrane fluidity and enhance the uptake of solutes like *gbu*. The presence of RNA helicases and cold-shock proteins (CspA) allows the bacterium to maintain gene expression and continue growing even at low temperatures [101,102,103]. Quereda et al. [5] further emphasize that cold-shock proteins may play additional functional roles beyond temperature stress response. *L. monocytogenes* is also frequently exposed to antimicrobials in POAO, food processing environments, or during sanitation procedures [5,58]. Sanitization processes are often not homogeneous and may fail to reach all areas effectively [104]. This leads to spatial variations in biocide concentration, creating “biocide concentration gradients” that allow pathogens to survive in certain locations where suboptimal disinfectant levels are present [104]. These subinhibitory concentrations are strongly associated with the development of tolerance mechanisms, enabling pathogens to acquire varying degrees of resistance to different disinfectants and antimicrobials, compromising effective microbial control [104]. One of the most commonly used disinfectant classes is quaternary ammonium compounds (QACs). However, certain *L. monocytogenes* strains have developed adaptive mechanisms, including efflux pump systems that actively expel toxic compounds, resulting in resistance to QACs such as benzalkonium chloride [105,106,107,108].

#### 3.1.2. Survival in Acidic Environments

Once ingested through the consumption of contaminated POAO, *L. monocytogenes* is exposed to hydrochloric acid (HCl), which maintains gastric pH and supports the digestive process [5]. In this environment, the pathogen encounters extremely acidic conditions (pH ~ 1–2), representing the host’s first physiological barrier—classified as a physicochemical barrier [5]. In addition to pH, several protective mechanisms contribute to blocking *L. monocytogenes* in the gastric environment, such as gastric motility, mucus production, and the epithelial barrier [22]. Therefore, individuals with altered gastric pH are more susceptible to *L. monocytogenes* infection, as reported by Jensen et al. [109], who detected the pathogen in the feces of patients undergoing chronic H2 antagonist treatment. HCl compromises bacterial structural integrity by promoting protein denaturation and triggering mechanisms that acidify the cytoplasm [22]. Moreover, gastric peristalsis itself acts as an additional mechanism to eliminate potentially harmful pathogens [22]. Nevertheless, *L. monocytogenes* employs various mechanisms to regulate its internal pH as a strategy to withstand gastric acid stress and acidic food environments [22,110].

One key acid resistance mechanism employed by *L. monocytogenes* is the glutamate decarboxylase (GAD) system [5]. This system involves the uptake of glutamate via the antiporter GadT, which is then decarboxylated to γ-aminobutyric acid (GABA) by the enzyme GadD. This reaction results in the consumption of a proton (H^+^), thereby raising the cytoplasmic pH [5]. GadT simultaneously exports GABA out of the cell in exchange for extracellular glutamate [5]. This process stabilizes the intracellular pH, enhancing bacterial survival under acidic conditions. Wemekamp-Kamphuis et al. [111] identified key components of this system, including transporters GadT1 and GadT2 and decarboxylases GadD1, GadD2, and GadD3. Additionally, the alternative sigma factor SigB plays an essential role in acid adaptation [111]. Another acid resistance mechanism involves the arginine deiminase (ADI) system, which contributes to maintaining intracellular homeostasis [5]. Within the cytosol, arginine is converted into ornithine via enzymes encoded by the *arcABC* operon, a gene cluster organized sequentially within the bacterial genome [5]. An antiporter encoded by *arcD*, known as ArcD, facilitates the exchange of intracellular ornithine for extracellular arginine [5]. As noted by Quereda et al. [5], “conversion of arginine to ornithine generates ammonia (NH_3_), which associates with a proton to produce NH_4_^+^, thus increasing cytoplasmic pH”. The genes within the *arcABC* operon are induced by exposure to acidic environments and arginine [112]. Chen et al. [113] reported that mutant strains lacking *arcB* exhibit reduced growth and survival under acid stress conditions. Furthermore, evidence indicates that ArgR, a transcriptional regulator, plays a role in the regulation of this pathway. However, further studies are needed to fully elucidate how the ADI system is regulated by pH, arginine availability, and SigB [114].

#### 3.1.3. Effects of the Pathogen on the Gut Endogenous Microbiota

The complexity of the intestinal environment imposes multiple adverse conditions for bacterial persistence, including “colonization resistance.” This protective phenomenon describes the microbiome’s ability to prevent the adhesion and growth of pathogens within the gastrointestinal tract, primarily driven by bacterial competition [5,22]. For the pathogen to establish its pathogenicity, bacterial translocation, bloodstream dissemination, and competition against the intestinal microbiota are necessary [22,72]. Several mechanisms contribute to colonization resistance, such as inhibiting enteric pathogen growth through competition for space and nutrients, immune system enhancement, and/or strengthening the intestinal barrier via the production of short-chain fatty acids [5]. The intestine presents multiple pathogen barriers, including the immune system and the mucus layer [22]. However, Rolhion and Chassaing [115] report that enteric pathogens have evolved subversive adaptations to host defense mechanisms, activating alternative pathways such as ethanolamine catabolism for nitrogen production via phosphatidylethanolamine, gastrointestinal inflammation potentiation against various agents, or the synthesis of bacteriocins (proteinaceous toxins). Pathogenesis relies on the pathogen’s ability to overcome the endogenous microbiota and evade immune destruction [22]. Consequently, the pathogen induces inflammation at the infection site [22]. The intestinal microbiome can counteract the pathogen by producing antimicrobial substances, altering local pH, and controlling infection [22].

Certain bacterial groups, such as *Lactobacillus* and *Clostridium* species, have been described as beneficial against *L. monocytogenes*. Archambaud et al. [116] demonstrated that pathogen-free mice pre-colonized with *Lactobacillus* species prior to *L. monocytogenes* exposure showed altered gene expression profiles in both the pathogen and host, resulting in restricted *L. monocytogenes* dissemination. Regarding *Clostridium*, Becattini et al. [117] showed that pre-inoculation impaired *L. monocytogenes* colonization in the intestinal tract and subsequent systemic spread. This study underscores the importance of microbiome diversity, including the potential use of probiotics as a preventative strategy in immunocompromised individuals and pregnant women. One aspect to consider is the ability of *L. monocytogenes* to re-adapt its nitrogen acquisition via alternative metabolic pathways, as confirmed by Archambaud et al. [116]. These findings highlight the challenges in controlling the pathogen due to its capacity to circumvent host-imposed “barriers.”

#### 3.1.4. Gut Barrier

In the intestinal region, the pathogen employs various strategies to ensure its penetration into the epithelium, facilitating entry into mesenteric lymph nodes and systemic dissemination [72]. The pathogen utilizes “three different main routes: (i) transcytosis, mainly through the invasion of goblet cells, and to a lesser extent, of enterocytes located in the tip of villi; (ii) para-cellular translocation, involving *Listeria* adhesion protein (LAP); and (iii) through M-cells into the Peyer patches” [5]. In route (i), for cells to be invaded, the interaction between InlA and E-cadherin (host receptor) is required [118,119]. E-cadherin facilitates phosphorylation and ubiquitination following binding to InlA, leading to clathrin-dependent endocytosis and nucleated actin polymerization [118,119]. Thus, for Nikitas et al. [118] and Kühbacher et al. [119], this process allows the pathogen to invade host cells, move intracellularly, and, depending on conditions, evade the immune system, ensuring cellular entry. Regarding route (ii), this occurs at the apical portion of the villi and depends on the interaction between LAP and its receptor (Hsp60) [5]. Although LAP is present in various species, it facilitates interaction only in pathogenic strains due to higher expression and secretion levels [120,121]. Upon LAP-Hsp60 binding, nuclear factor kappaB (NF-κB) is activated, triggering the secretion of pro-inflammatory cytokines (e.g., interleukin-6) and myosin light chain kinase (MLCK), which is responsible for structural and functional alterations of epithelial or endothelial tight junctions [122]. Consequently, the pathogen translocates from the luminal space into the lamina propria [122]. The third route (iii) involves Peyer’s patches, lymphoid tissue aggregates containing M cells. These cells capture antigens for presentation to macrophages, constituting an intestine-specific immune mechanism (GALT) [5].

It is worth noting that the pathogen is capable of invading and proliferating within both phagocytic and non-phagocytic cells [5]. The entry of *L. monocytogenes* is mediated by interactions between internalins, especially InlA and InlB, and their receptors. Moreover, ActA and LLO also contribute to this internalization [72,123]. After cellular entry, the pathogenicity island 1 encodes virulence factors that enable escape from endosomes or phagosomes, cytosolic proliferation, and cell-to-cell dissemination [22,123,124,125]. The pathogen employs specific mechanisms to target LLO activity to the vacuolar membrane, causing pore formation exclusively in these membranes [5]. LLO exhibits effective activity at the acidic pH of vacuoles, which preserves the host cell membrane integrity, as the neutral cytosolic pH leads to LLO degradation [126]. LLO activity is further reduced in the cytosol by mechanisms such as oxidoreductases [127]. Generally, the mechanisms employed by the pathogen involve cellular displacement, facilitated entry via internalins, propagation, and immune evasion through LLO and phospholipases [22]. Thus, Quereda et al. [5] and Oliveira et al. [22] emphasize that these processes allow the pathogen to disrupt vacuolar membranes, survive within the cytosol, and disseminate to other cells without immune recognition. The authors note that if the pathogen were to destroy the host cell membrane, it would be readily detected and eliminated by the immune system. A feedback loop based on messenger RNA tightly regulates LLO activity in intracellular pathogens [128,129,130]. Therefore, the pathogen exhibits abilities for intestinal cellular adhesion and invasion, immune modulation and evasion, as well as favorable intracellular growth [22].

#### 3.1.5. Adaptation to Osmotic Stress and Bile Salts in Listeria Monocytogenes

The intestinal region presents variations in osmolality that assist *L. monocytogenes*, with certain sites exhibiting higher solute concentrations [5]. In such conditions, for example during osmotic stress, the pathogen focuses on acquiring solutes by expressing transporters such as *gbu* and *betL* [110]. It is also important to highlight that, depending on osmoprotection-associated proteins like proteases and proline synthases, cross-protection and pre-adaptation to other types of stress may occur [99,131]. One of the host’s natural antimicrobial agents is bile, composed of products such as bile acids and biliverdin [22]. Bile acids are produced and stored in the gallbladder to be released during digestion, assisting in cholesterol elimination [132]. These acids contribute to bacterial control by increasing oxidative cytosolic stress on the pathogen, as well as causing cellular damage through mechanisms like protein unfolding [132]. Furthermore, bile may serve as an extracellular replication site for the pathogen [133], due to its more basic pH compared to the stomach, facilitating dissemination to the intestinal region. The survival of *L. monocytogenes* in the presence of bile may be related to bile salt hydrolase activity; after bile salt deconjugation, toxicity toward the pathogen is reduced, with maximal activity in the duodenum, where bile pH decreases [72,134]. Additionally, bile tolerance may be linked to the membrane transporter BilE, which expels bile salts that enter the cell, thus preventing cellular damage and protein destabilization [72,135]. Evidence suggests that secondary bile salts influence bacterial regulation, and in *L. monocytogenes*, resistance to bile involves regulation by SigB and PrfA. Exposure to bile induces expression of virulence genes, with a shift from SigB to PrfA expression [136,137,138,139].

#### 3.1.6. Other Pathways of Infection

Evidence describes the pathogen’s role in various organ systems such as blood, immune system, liver, spleen, brain, and placenta. In immunocompromised hosts, pathogen dissemination occurs through the lymphatic system and bloodstream, targeting the liver and spleen [140]. A small portion of *L. monocytogenes* is intracellular, efficiently spreading to other lymphoid organs such as the spleen and lymph nodes, as well as the liver; the remaining gastrointestinal population is extracellular [141]. In guinea pigs, hepatic distribution of *L. monocytogenes* occurs via two routes: the first through the intestine-portal vein-liver axis, and the second via mesenteric lymph nodes, reaching the bloodstream and disseminating to the liver and spleen [142]. Quereda et al. [5] describe that “*L. monocytogenes* circulates in blood either freely or associated with mononuclear phagocytes and polymorphonuclear leukocytes”. In the bloodstream, virulence is regulated by PrfA, whereas in the intestine it is mediated by SigB [143]. Moreover, when the pathogen is in the blood circulation, *L. monocytogenes* is associated with cellular remodeling, specifically at the surface level, with increased levels of InlA and LAP, for example [144], while others such as internalins I show negative regulation [145]. This surface remodeling process enables the pathogen to survive bactericidal action in blood and plasma and ensures systemic involvement [5].

Regarding the immune system, the pathogen triggers innate immunity, which initially helps control bacterial load and growth restriction [5,59]. At the end of this process, there is a specific lymphoid response aimed at eliminating the infection and establishing immune memory [59]. However, as an adaptive trait, *L. monocytogenes* evades the host immune system through various strategies [59]. One such strategy involves utilizing actin-based motility to spread without exposure to the extracellular environment, thus evading classical immune responses and being affected mainly by CD8+ cell populations [59]. Another strategy involves internalins C (InlC), which, for to Gouin et al. [146], are secreted by the pathogen and interact with IκB kinase (IKKα) to release the NF-κB transcription factor responsible for inflammation. *L. monocytogenes* blocks NF-κB activation by inhibiting IKKα, thereby impairing inflammatory responses, neutrophil chemotaxis, and cytokine activation [146]. Lebreton et al. [147] further report that nucleomodulins secreted by the pathogen can induce epigenetic changes favorable to bacterial survival. Additionally, Cossart [140] and Boneca et al. [148] highlight that alterations in the bacterial cell wall contribute to resistance and evasion of innate immune responses.

From a hepatic perspective, *L. monocytogenes* infection leads to apoptosis and necrosis of Kupffer cells during early infection, inducing inflammation followed by liver repair involving monocyte recruitment and differentiation into macrophages for tissue replacement [149]. Wang et al. [150] emphasize that hepatocytes also recruit monocytes via Toll-like receptor 2, stimulating chemokines such as CCL2 and CXCL1 to attract monocytes and neutrophils, which may help contain infection spread. In the spleen, the pathogen is restricted by innate immune mechanisms aided by dendritic cells and macrophages [151]. Dendritic cells activate CD8+ lymphocytes during chronic phases to eliminate the pathogen and serve as sentinels supporting adaptive immunity [152]. Dendritic cells may also act as reservoirs for the pathogen, enabling immune evasion and access to the mononuclear phagocyte system [5].

Numerous studies indicate *L. monocytogenes* has a particular tropism for the central nervous system (CNS) in hosts such as humans and ungulates [153,154]. Koopmans et al. [72] describe the neuropathogenesis involving the blood–brain or blood–cerebrospinal fluid barriers, though the exact site remains unclear. The neuroinvasion is attributed to the pathogen’s ability to cross the blood–brain barrier (BBB) and to axonal transport to neuronal cell bodies [5]. For Quereda et al. [5], “retrograde axonal transport occurs through two different routes: (i) one that utilizes the cranial nerves—primarily the trigeminal nerve—upon crossing of the oral epithelium; and (ii) one which exploits the olfactory epithelium.” In the first route, *L. monocytogenes* enters through oral mucosal lesions affecting cranial nerves, more frequently observed in ruminants [154,155]. This mechanism is associated with rhombencephalitis, as noted by Oevermann et al. [76]. Neonates may develop neurolisteriosis via the olfactory epithelium route at birth [156]. Additionally, *L. monocytogenes* can cross the BBB by recognizing surface receptors, possibly involving leukocytes, facilitating CNS access [154,157,158]. For the authors, this leads to meningitis and meningoencephalitis development. Quereda et al. [5] state, “three virulence factors from LIPI-1 play a role during brain infection: PlcB, LLO, and ActA”. Furthermore, evidence suggests internalins InlA and InlB contribute to brain infection [157]. Maury et al. [159] note strain-dependent differences in virulence, with some exhibiting higher neurotropism.

The role of *L. monocytogenes* in the placenta can be described through two pathways: one involving maternal infected phagocytes disseminating the pathogen, and another through direct contamination of trophoblasts by bloodborne bacteria, with pathway selection depending on factors such as hepatic/splenic pathogen load and inoculum dose [160]. Thus, dissemination is hematogenous [72]. Quereda et al. [5] describe the placental barrier as composed of cytotrophoblasts, precursors to syncytiotrophoblasts, which directly contact maternal blood, along with extravillous cytotrophoblasts involved in placental attachment to the uterus—both susceptible to invasion [161]. Koopmans et al. [72] identify the cytotrophoblast region as the preferred site of infection. Additionally, InlA and InlB are critical for placental infection, with InlP necessary for bacterial entry and traversal of epithelial cells [162,163]. Cases of *L. monocytogenes* infection in the maternal–fetal axis are linked to clonal complexes with high virulence [5].

#### 3.1.7. Microbial Biofilms

Biofilms are characterized as communities of microorganisms that adhere to different surfaces with a defined structure [164]. Zawiasa et al. [165] reported that different pathogenic species, such as *L. monocytogenes* and *L. innocua*, can coexist; however, the former demonstrates superior adhesion ability in pure culture. They are typically composed of a self-produced extracellular polymeric substance (EPS) matrix, characterized by a network of polysaccharides, proteins, nucleic acids, and other compounds secreted by the microorganisms themselves, which function to encapsulate and adhere the biofilm to surfaces [164]. The presence of the EPS matrix enables pathogens like *L. monocytogenes* to protect themselves from environmental stressors and antimicrobials, allowing for attachment, structural organization, and internal communication within the biofilm [22,164]. A comprehensive overview of EPS is available in the review article by Tuytschaever et al. [166], and other studies published on biofilm control may be useful for a better characterization of the situation [14,167]. It is worth noting that although biofilms are often viewed as harmful, their presence can be beneficial in certain situations, such as fermentation processes [164]. Controlling biofilms can be particularly challenging, especially when associated with highly virulent pathogens. As stated by Coppola et al. [164], “interventions targeting harmful biofilms may unintentionally disrupt beneficial communities, while their dynamic and adaptive nature further challenges selective control”. Overall, biofilms can be considered strategies for protection, dissemination, and persistence of microbial agents. For Diaz et al. [168], the pathogen can benefit from the symbosis between the microbiological community. Voglauer et al. [169] highlight that coexistence enables resistance to elimination, thereby aiding in the pathogen’s dissemination.

Biofilm formation involves the development of distinct stages: initial attachment, transition to sessile cells, maturation, and dispersion. The attachment stage involves the pathogen aggregating into microcolonies once it exceeds a certain biomass threshold, detaching, and adhering to a new surface [170,171]. For Coppola et al. [164], “up to this point, it is still relatively easy to remove the adhered cells”. After initial adhesion, planktonic cells transition into sessile cells through cellular modifications that lead to EPS production [170,171]. EPS allows these cells to be protected, encapsulated, adhered, and capable of colonization and proliferation into microcolonies [170,171]. Communication between microcolonies occurs through quorum sensing (QS) [82,172]. For Coppola et al. [164], “through the production and detection of small signaling molecules (autoinducers), QS enables microbial populations to sense cell density and coordinate gene expression collectively”. QS also supports processes such as EPS production, motility, virulence factor expression, and adaptation to stress conditions [170,171]. One advantage of QS is its role in reducing the efficacy of antimicrobials, as well as enhancing adaptive capacity under diverse environmental conditions [170,171]. As emphasized by Coppola et al. [164], “disrupting QS pathways represents a promising strategy to interfere with this synchrony, attenuate biofilm robustness, and sensitize microbial populations to antimicrobial agents or environmental stressors”. Furthermore, in addition to having a fundamental role in biofilms, Meireles et al. [82] state “Lm QS systems have been associated with bacterial communication and survival providing competitive advantages in adhesion to surfaces, biofilm formation, invasion of mammalian cells, mouse infection, and changes in gene expression”. This explains the potential role of QS, beyond biofilm formation, in the dynamics of bacterial survival, adaptation, and pathogenesis.

The three-dimensional structures involved in nutrition and waste management within biofilms allow sessile cells to detach, revert to planktonic forms, and colonize new surfaces [173,174]. Industrial environments often harbor a wide variety of bacterial communities, particularly those with high pathogenic loads [164]. The composition of the biofilm matrix is diverse, containing polysaccharides, proteins/enzymes, extracellular DNA (eDNA), and lipids, which globally function to ensure adhesion, cohesion, and signaling [175,176,177,178,179]. Coppola et al. [164] states, “the biofilm exhibits a stratified arrangement: a superficial layer, populated by metabolically active cells, with access to oxygen and nutrients; an intermediate layer, densely populated, with less metabolically active bacteria and a higher accumulation of EPS; and a deep layer characterized by cells in a quiescent or dormant state, with limited availability of oxygen and nutrients”. The author also highlights that the deepest layer, composed of quiescent cells, allows these cells to be less susceptible to antimicrobial activity and enables the persistence of the biofilm. Antimicrobial resistance in bacterial pathogens within biofilms results from adaptive processes aimed at survival and persistence, compromising food production facilities handling POAO [164]. For Arthur et al. [180], “key factors including strain type, surface characteristics, incubation temperature, biofilm age, presence of organic matter, mono- or multispecies biofilm, biocide’s active ingredients and concentration, contact time, and detachment and enumeration methods play pivotal roles in biofilm response and need to be considered in assessing the efficacy of a biocidal product”. *L. monocytogenes* is capable of overcoming sanitation protocols, surviving refrigeration, persisting in POAO processing environments, and contaminating products through cross-contamination [164]. Coppola et al. [164] emphasizes that this ability underscores the importance of implementing stricter hygiene programs focused on biofilm removal.

## 4. Detection Methods, Control Strategies, and Brazilian Legislation

Considering the broad impact of *L. monocytogenes* on both human and animal health, detection methods and control strategies are essential to contain the spread of the pathogen and reduce contamination rates among exposed populations. However, Vallim et al. [181] emphasize that the costs and methodologies involved in pathogen identification represent significant obstacles that may contribute to inaccurate prevalence data, directly affecting food safety monitoring and epidemiological surveillance. For Vasileiadi et al. [27], “without effective prevention and control measures, Lm constitutes a potential risk for cross-contamination in the food chain”. Various diagnostic methods can be employed, including enrichment and isolation, molecular techniques, and other emerging approaches. For Ravindhiran et al. [3], “according to the standard procedures, isolation of *L. monocytogenes* from samples to enrich the culture of the CFU/mL within the range of 10^4^–10^5^ in each 25 g of the sample”. The ISO 11290-1:2020 standard, currently enforced by the Brazilian Association of Technical Standards (ABNT) [182], establishes that detection involves four steps: (1) primary selective enrichment in Demi-Fraser broth, incubated for 24–26 h at 30 °C; (2) secondary enrichment in Fraser broth for 24 h at 37 °C; (3) plating; and (4) colony identification. Meanwhile, ISO 11290-2:2020 [183] outlines enumeration of the pathogen in five steps: (1) initial suspension in an appropriate diluent; (2) surface plating on a specific medium; (3) incubation in Petri dishes at 37 °C with two readings—after 24 h and 48 h; (4) colony confirmation; and (5) enumeration expressed in grams per milliliter, square centimeter, or equipment-based sampling. Both procedures require two enrichment steps, followed by confirmation using biochemical tests such as Gram staining, catalase, motility, and sugar fermentation, among others. Overall, these steps may take up to six days to yield a final result [184,185,186]. The full methodology is available through the ABNT website under ISO 11290-1:2020 and ISO 11290-2:2020; however, the documents are behind a paywall.Another detection method is based on the protocol developed by the Food and Drug Administration (FDA) through the Bacteriological Analytical Manual (BAM) [186]. This method requires enrichment in Buffered *Listeria* Enrichment Broth (BLEB) for 24–48 h, followed by plating on esculin agar [186]. Colonies with typical morphology are then subcultured on TSAYE, with subsequent biochemical confirmation. For to Hitchins et al. [187]: “1. Incubate food samples or environmental samples homogenized in basal BLEB (M52, 43) at 30 °C, for 4 h. 2. Aseptically add the three filter sterilized selective agents (M52) to achieve final concentrations of 10 mg/L acriflavin, 50 mg/L cycloheximide and 40 mg/L sodium nalidixic acid in the BLEB pre-enrichments. 3. Mix the enrichment with additives and continue incubation at 30 °C for the remainder of the 24 to 48 h enrichment period”. The complete BAM protocol is available free of charge on the FDA’s official website.

Another detection method is based on the Association of Official Analytical Chemists (AOAC), which describes analysis through Loop-Mediated Isothermal Amplification (LAMP) following enrichment in demi-Fraser broth, DNA release by lysis, LAMP amplification, and result reading within 24 h [188]. The complete process can be found in the Official Methods of Analysis of AOAC INTERNATIONAL (22nd Edition). The ISO, BAM, and AOAC methods are considered gold standards for diagnosing *L. monocytogenes* [121]. A major challenge in detecting *L. monocytogenes* lies in the species diversity, which means no validated and fully efficient method exists [189]. Conventional methods are generally effective and straightforward but are costly and time-consuming [3]. The delay in obtaining results can be a significant obstacle, thus earlier detection methods based on immunology, spectrometry, and molecular techniques, among others, are necessary [20,190,191,192,193]. Jadhav et al. [190] also mention other drawbacks of conventional methods, including: “extensive reliance on phenotype which is subject to changes under different environmental conditions; requirement for different chemicals, media and reagents; interference due to contaminating bacteria which can mask the presence of the target organism; and atypical reactions given by atypical strains”. A comprehensive review of diagnostic methods for *L. monocytogenes* can be found in Gasanov et al. [194], which includes a flowchart of detection steps and available options.

For Dincer [20], “enzyme-linked immunosorbent assay (ELISA), enzyme-linked fluorescent assay (ELFA), thermal flow immunoassay and immunomagnetic separation are among the most used immunological methods. Simple polymerase chain reaction (PCR), multiplex PCR, real-time PCR (RT-PCR), real-time nucleic acid sequence-based amplification, oligonucleotide-based microarray, and loop-mediated isothermal amplification are among the most used molecular detection methods”. Other approaches are based on spectroscopy and spectrometry [20,190], as well as sensor-related technologies [192,193,195]. Immunological assays are valid but detect whole cells, are less sensitive compared to amplification methods, and have disadvantages such as time consumption, DNA viability issues, and cross-reactivity [190]. ELISA allows antibody immobilization and is simplified but also shows reduced diagnostic sensitivity [190]. The same author states that magnetic separation using paramagnetic polystyrene beads is valid diagnostically but only for viable cell groups. Regarding molecular methods and other techniques such as PCR, DNA microarrays (which use probes to detect bacterial groups or polymorphisms), multiplex PCR (superior to conventional PCR for detecting multiple pathogens), biosensors (biomolecular interactions), and LAMP amplification (DNA amplification) have also been proposed as valid diagnostic methods [3,24,190,196,197,198,199,200]. The multiplex PCR described by Rawool et al. [201] was considered a more simplified and cost-effective way to detect the pathogen and its strains. It is important to note that all methods have their advantages and disadvantages, including the possibility of false positives or negatives [20]. Rawool et al. [201] emphasize that developing a method that is efficient, accessible, rapid, and capable of processing many samples is essential for identifying *L. monocytogenes* and its strains. This would deepen understanding of the microorganism’s genetic diversity, ecological dynamics, and dissemination patterns [201]. The authors also discuss that integrating species identification with molecular subtyping tools would greatly speed up monitoring and control efforts, especially for variants associated with outbreaks of foodborne diseases. Given the impact of the disease and the increasing demand from consumers for high-quality products, the food industry has been exploring the use of natural antimicrobials as a strategy to extend shelf life and ensure food safety [9,202]. Regarding *L. monocytogenes*, Papadochristopoulos et al. [9] highlight the potential application of chitosan for bacterial control in beef hamburgers and Tu et al. [202] about the use of *Aspergillus oryzae*; however, further evaluations are still needed to validate the effectiveness of this approach. Other options such as nanoparticle-based food packaging films for *L. monocytogenes* antimicrobial control are provided in the review by Furlaneto and Furlaneto-Maia [203]. Table 1 summarizes the principal diagnostic methods for *L. monocytogenes*, outlining their key advantages and limitations as reported in the literature.

Regarding Brazilian legislation, Normative Instruction No. 9/2009 (IN No. 9 of 2009), dated 8 April 2009, from the Ministry of Agriculture, Livestock, and Supply (MAPA) [204], in the annex entitled “Procedures for Control of *Listeria monocytogenes* in Ready-to-Eat Animal-Origin Products”, aims to establish the microbiological limits of *L. monocytogenes* in POAO, as a means to ensure food safety. However, the IN does not constitute an epidemiological surveillance instrument, as it does not highlight the need for mandatory notification of human cases nor the importance of monitoring positive isolates. Therefore, supplementary strategies need to be taken, such as outbreak investigations, molecular screenings/sequencing, and integration with laboratory surveillance, so that the investigation of its presence becomes more effective. The IN No. 9/2009 states:

“Art. 1° The control procedures for *Listeria monocytogenes* in ready-to-eat animal-origin products aim to monitor and ensure the safety of these products concerning this pathogen and apply to establishments that manufacture animal-origin products.”

“Sole Paragraph. This regulation applies to establishments manufacturing ready-to-eat animal-origin products with the following physicochemical characteristics: pH > 4.4 or Water Activity (Aw) > 0.92 or sodium chloride concentration < 10%, respecting their production process characteristics.”

Regarding inspection, Chapter III of IN No. 9 of 2009, titled “Inspection and Official Verification of the Control Procedure for *Listeria monocytogenes* in Ready-to-Eat Animal-Origin Products,” establishes:

“Art. 6° Positive results for *Listeria monocytogenes* will trigger inspection procedures of the production process and review of records for ready-to-eat animal-origin products.”

“§ 1° The inspection of the production process must cover the following aspects:

I—evaluation of facilities and equipment aimed at preventing cross-contamination;

II—evaluation of the ease of cleaning equipment used;

III—evaluation of hygienic-sanitary habits and personal hygiene of employees;

IV—evaluation of raw material conditions and technological procedures in producing ready-to-eat animal-origin products;

V—evaluation of methods used by the establishment to reduce biological contamination of packaged ready-to-eat animal-origin products;

VI—evaluation of methods used by the establishment to suppress microorganism multiplication in packaged ready-to-eat animal-origin products.”

“§ 2° The review of production process records must include:

I—evaluation of compliance with self-control procedures’ results, emphasizing monitoring of controls applied, including preventive measures, corrective actions, and microbiological tests;

II—evaluation of the authenticity of the records.”

IN No. 9 of 2009 also states:

“Art. 7° Official inspection results must be clearly and thoroughly recorded, citing all records that motivated the enforcement action as well as the specific legislation.”

“Art. 8° When corrective and preventive measures implemented are deemed ineffective, restrictive actions must not be lifted until proven adequate alternatives are presented.”

“Art. 9° Ready-to-eat animal-origin products positive for *Listeria monocytogenes* may be reprocessed, provided the applied procedure ensures microorganism destruction.

§ 1° After reprocessing, establishments must perform microbiological analysis confirming absence of *Listeria monocytogenes*.

§ 2° If reprocessing is not feasible or does not guarantee elimination of the microorganism, the products must be destroyed.”

“Art. 10° When *Listeria monocytogenes* is detected in ready-to-eat animal-origin products, inspected establishments must review their self-control procedures.”

“§ 1° The review of self-control procedures should focus on:

I—control of raw materials, ingredients, and primary packaging;

II—production processes aiming to reduce contamination levels of ready-to-eat animal-origin products;

III—sanitation and cleaning programs to reduce biological contamination, focusing on *Listeria monocytogenes* during production;

IV—control of environment and equipment to prevent recontamination after product manufacture;

V—adequate methods to reduce biological contamination in packaged ready-to-eat animal-origin products;

VI—adequate methods to suppress microorganism multiplication in packaged ready-to-eat animal-origin products.”

“§ 2° Inspected establishments must:

I—establish records demonstrating the effectiveness of implemented self-control programs;

II—implement monitoring of the processing environment for Listeria monocytogenes or *Listeria* spp.”

“Art. 11° If, during official inspection, it is found that the establishment where *Listeria monocytogenes* was detected has not implemented the actions provided herein, the following measures must be taken:

I—product seizure;

II—microbiological tests for Listeria monocytogenes before releasing the product for consumption.”

“Sole Paragraph. Measures imposed on the establishment will only be lifted after confirmation of implementation of the provisions in Art. 10 of this annex.”

Regarding the Normative Instruction N° 161, dated 1 July 2022, based on the Collegiate Board Resolution (RDC) N° 724, also dated 1 July 2022, from the Brazilian Health Regulatory Agency (ANVISA) [205], concerning microbiological standards for food:

“Art. 4° Annex II establishes microbiological standards for *Listeria monocytogenes* in ready-to-eat foods.”

“Sole Paragraph. The following foods are exempt from regular *Listeria monocytogenes* testing if they meet at least one of the following conditions:”

“I—shelf life less than 5 days;

II—pH less than or equal to 4.4;

III—water activity less than or equal to 0.92;

IV—pH less than or equal to 5.0 and water activity less than or equal to 0.94;

V—products that received effective thermal treatment or equivalent process eliminating Listeria monocytogenes and where recontamination after treatment is not possible, such as products thermally treated in their final packaging;

VI—fresh, whole, unprocessed fruits and vegetables, excluding sprouted seeds;

VII—breads, cookies, and similar products;

VIII—bottled waters, carbonated waters, soft drinks, beers, ciders, wines, and similar products;

IX—sugars and sweetening products;

X—honey;

XI—chocolate and cocoa products;

XII—candies, chocolates, and chewing gums;

XIII—live bivalve mollusks.”

In Annex II—Microbiological Standard for *Listeria monocytogenes* in ready-to-eat foods, the following apply:

n—number of sample units;

c—maximum allowable number of sample units;

m—maximum acceptable value per sample;

M—unacceptable value per sample.

It establishes:

(a) Ready-to-eat foods (except for infants or special purposes): *Listeria monocytogenes*: n = 5, c = 0, m = 10^2^ CFU/g or mL, M = —.
(b) Ready-to-eat foods intended for infants or special purposes: *Listeria monocytogenes*: n = 10, c = 0, m = absence in 25 g or mL, M = —.

## 5. Overview of Listeria Monocytogenes in Brazil’s Public Health Context

As an opportunistic pathogen associated with biofilm formation and the presence of strategies to evade the host immune system, *L. monocytogenes* stands out as a significant bacterial agent that poses a threat to animal health and, particularly, human health. As previously noted by Ravindhiran et al. [3], Meireles et al. [82] and López et al. [206], immunocompromised individuals are more susceptible, including the elderly (over 65 years), pregnant women, infants and children under five years old, and patients with diseases such as Human Immunodeficiency Virus (HIV). Thus, the social impact of this pathogen is evident, causing outcomes ranging from mild to severe, which may ultimately result in fatal cases. To control this and ensure that POAO continue to meet strict nutritional and microbiological standards, processing establishments must prioritize product quality from raw material to commercialization, instituting control programs, operational procedures, proper sanitation, and disposal of potentially contaminated materials that pose risks to human health.

Within the One Health context, various factors have been linked to the rising incidence of *Listeria* spp. cases, as reported by Quereda et al. [5]: “(i) the increasing susceptible population: aged and immunosuppressed patients (HIV, cancer or transplant patients); (ii) industrialization of food production and the subsequent risk of large distribution of contaminated food; (iii) the generalization of food preservation methods, such us refrigeration, which allows *L. monocytogenes* selective growth; (iv) increased consumption of preservative-free RTE foods; (v) use of antacids and gastric-acid-suppressive medications; and (vi) improved diagnostic methods and enhanced public health surveillance”. Thus, the population becomes vulnerable to the presence of the pathogen, which is persistent and difficult to control. In some countries, the occurrence of the pathogen overwhelms healthcare services, becoming a significant threat to the population. It is important to highlight that the severity of the effects resulting from *L. monocytogenes* contamination is exacerbated in immunocompromised patients, making them particularly susceptible hosts. This issue is compounded by factors such as limited urban infrastructure, restricted access to safe drinking water, low-quality food, and inadequate basic health services. In Brazil, the population is frequently exposed to precarious living conditions, residing in substandard housing near rivers, in densely populated urban areas, and with limited access to essential infrastructure. As a result, these groups are more potentially exposed to the disease. Therefore, control and prevention strategies to limit the presence of L. monocytogenes are increasingly necessary to prevent its persistence and dissemination in food processing environments, in areas with low infrastructure, and among more vulnerable populations.

In Brazil, the disease currently presents as sporadic and isolated cases, unlike outbreaks described in countries such as the United States and Europe. Notably, the disease is not listed among mandatory notifiable conditions under Portaria de Consolidação GM/MS n° 4, dated 28 September 2017 [207], amended by Portaria GM/MS N° 6.734, dated 18 March 2025 [208], which hinders accurate knowledge of its occurrence. This is mainly due to its low reported incidence, the diagnostic challenges associated with its detection, and the current focus on outbreak investigations and high-risk groups rather than individual case reporting. Regulation occurs through the implementation of normative instructions and regulatory agencies [204,205]. Reis et al. [189] indicate underdiagnosis and underreporting within Brazil. However, Annex I of the National List of Compulsory Notification of Diseases, Conditions, and Public Health Events (Portaria GM/MS N° 6.734, 18 March 2025) [208] includes item 22 defined as “Public Health Event (ESP) that constitutes a threat to public health.” This condition is defined in Chapter I, Section I, Article 2, item 5 as “a situation that may constitute a potential public health threat, such as an outbreak or epidemic, disease or condition of unknown cause, changes in the clinical-epidemiological pattern of known diseases, considering dissemination potential, magnitude, severity, transcendence, and vulnerability, as well as epizootics or conditions resulting from disasters or accidents.” Thus, if the disease occurs as an outbreak and poses a potential public health threat, compulsory notification must occur within 24 h. Comparatively, the situation of the disease in regions such as Europe and the United States is also characterized by the occurrence of outbreaks, but with greater number and scope. Furthermore, these countries emphasize the mandatory notification of cases [209,210]. Another difference can be observed in the investigation process, which, as described by Lomonaco et al. [211], includes more thorough analyses of the genetic characteristics of each pathogen through sequencing. A point of similarity is based, according to Lomonaco et al. [211], on the statement that “several challenges are connected to the epidemiologic investigations of listeriosis clusters, such as a small number of geographically dispersed case-patients, the lengthy incubation period, the difficulty of patient store call food exposures, and the fact that patients may die”. This highlights that, even in different locations, the challenges related to disease investigation are present and play a determining role in its magnitude.

Considering the impact of the pathogen in Brazilian territory, the study by Camargo et al. [212] aimed to investigate the genetic variability of *L. monocytogenes*. The authors observed that the process of pathogen isolation in the described outbreaks is superficial, which hinders knowledge about it. It was noted that most isolates belonged to lineages I and II, with 10 distinct sublineages (highest prevalence of SL9, SL3, and SL2/SL218) and 31 new core genome multilocus sequence typing profiles. They also observed that some strains showed resistance and environmental persistence, being detected over intervals of several years. Some strains carried genes related to survival, all possessed LIPI-1, and a portion had LIPI-3 and LIPI-4.

In 2007, Mantilla et al. [213] analyzed 30 refrigerated beef samples from markets in Niterói, Rio de Janeiro, and found 50% contaminated with *Listeria* spp., with *L. monocytogenes* being the second most frequently detected, particularly serotype 4b. In 2013, Monteiro et al. [214], aiming to analyze pathogen strains in raw ground beef and sausage samples, observed approximately 17% contamination in ground beef and nearly 7% in sausage samples with various serotypes. Mendonça et al. [215] demonstrated that refrigerated chicken meat was contaminated (33.3%) with distinct genotypic profiles of the bacterium. Silva et al. [216] investigated *L. monocytogenes* presence in fresh beef samples sold in 10 butcher shops at a market in Bahia and found contamination in 50% of shops, with approximately 55% of meats contaminated. Soares et al. [217] showed that after analyzing nearly 240 meat product samples, the pathogen was most prevalent in fresh sausages. The same study found that nearly 44% of all products failed to meet microbiological standards. A systematic review by Cavalcanti et al. [218] on *L. monocytogenes* prevalence in Brazil over ten years revealed a combined prevalence of 13% (range 0–59%) using a random-effects model. For raw and ready-to-eat meats, combined prevalence was 14% and 11%, respectively. The authors noted higher prevalence in pork products and in the Southeast region, with greater occurrence in retail and diverse associated serotypes. The 2019 Annual Report of the Animal-Origin Food Control Programs and Animal Feed Products of DIPOA, issued by MAPA [219], state that *L. monocytogenes* prevalence was 0.96% among 1035 samples tested. In 2024, MAPA [220] reported 1.45% non-compliance in 967 samples analyzed, mostly meat products. Although prevalence remained under 2% in 2024, all suspected or confirmed cases require full investigation to avoid over- or underestimation of incidence. Furthermore, there is an increasing need for rigorous microbiological control during POAO processing, including proper temperature storage and the implementation of sanitization and hygiene protocols in industries and establishments.

## 6. Final Considerations

Given the above, the significant risk posed by FBDs originating from foods contaminated with *Listeria* species, especially *L. monocytogenes*, becomes evident. Considering the characteristics described in previous sections, it is important to highlight the pathogen’s ability to adapt in order to ensure its growth, development, and persistence. It is worth emphasizing that this bacterium can readily induce clinical syndrome and evade the host immune response. It is a foodborne pathogen of high importance due to its capacity to cause outbreaks with rapid dissemination and a wide range of clinical symptoms in both type and severity, particularly in patients with compromised immune systems such as the malnourished, those with poor nutrient absorption, and immunocompromised individuals including cancer patients and those with acquired immunodeficiency syndrome. Its dissemination can be controlled through the application of good manufacturing practices, proper storage, and effective sanitation. Currently, *L. monocytogenes* is characterized by its ease of biofilm formation, which facilitates its survival on equipment and facilities used in the processing of POAO. This represents a serious One Health challenge by promoting food contamination and compromising both quality and safety. Food safety is a critical topic because human consumption depends on foods with adequate nutritional and microbiological quality. The FBDs reduce product viability and have significant economic repercussions. Therefore, enhanced control measures, development, and implementation of good practices throughout the entire POAO production chain—from raw material sourcing to final consumption—are essential, with a focus on facility and equipment sanitization, as well as personal hygiene.

## Figures and Tables

**Table 1:** Methods for detection of *L. monocytogenes*: advantages and disadvantages, based in evidences published [3,20,27,181,184,185,186,187,188,189,190,191,192,193,194,201,202].

Diagnostic Method	Advantages	Disadvantages
Classical culture/ISO 11290-1 and 11290-2 (ABNT)	-Gold standard for isolation -Allows subsequent strain typing -Internationally standardized methodology	-Time-consuming (up to 6 days) -Requires two enrichment steps -Relatively high costs -Sensitive to competing contaminants -Produce live bacterial colonies
BAM/FDA	-Standardized method, freely available -Biochemical confirmation -Allows outbreak traceability	-Time-consuming (24–48 h enrichment + subsequent steps) -Requires prior isolation -Multiple and complex steps -Produce live bacterial colonies
AOAC/LAMP	-Rapid (results in 24 h) -High sensitivity -Can be applied after enrichment	-Requires specialized equipment -Requires DNA extraction -Does not produce live bacterial colonies
PCR/Multiplex PCR	-Rapid and specific -Multiplex allows detection of multiple genes or pathogens simultaneously -Faster than classical culture	-Does not allow bacterial isolation -Sample inhibitors possible -Cost of reagents -Does not produce live bacterial colonies
qPCR/RT-PCR	-Quantitative detection -High sensitivity -Faster than culture-based methods	-Requires calibration and controls -Does not produce live bacterial colonies -Expensive equipment
DNA Microarrays/Biosensors	-Allows analysis of multiple targets simultaneously -Useful for environmental or food monitoring	-High cost -Requires specialized infrastructure -Complex data interpretation -Does not produce live bacterial colonies
ELISA/ELFA/Immunomagnetic separation	-Simple to use -Detects specific antigens or viable cells	-Lower sensitivity compared to PCR -Time-consuming -Possibility of cross-reactivity -Does not produce live bacterial colonies
Spectroscopy/Spectrometry	-Emerging rapid methods -Potential for direct analysis in food	-Methods still under development -Expensive equipment -Requires robust validation -Does not produce live bacterial colonies

## Data Availability

No new data were created or analyzed in this study. Data sharing is not applicable.

## Abbreviations

§	Paragraph
°C	Degrees Celsius
ABNT	Brazilian Association of Technical Standards
ActA	Actin assembly-inducing protein
ADI	Arginine deiminase
ALOA	Listeria Ottaviani and Agosti Agar
ANVISA	Brazilian Health Regulatory Agency
AOAC	Association of Official Analytical Chemists
Art.	Article
Aw	Water activity
BAM	Bacteriological analytical manual
BBB	Blood–brain barrier
*betL*	Gene encoding a glycine betaine
BLEB	Buffered Listeria enrichment broth
c	Maximum allowable number of sample units
CC	Bacterial clones
CFUs	Colony-forming units
CNS	Central nervous system
CspA	Cold-shock proteins
eDNA	Extracellular DNA
ELFA	Enzyme-linked fluorescent assay
ELISA	Enzyme-linked immunosorbent assay
EPS	Extracellular polymeric substance
ESP	Public health event
FBDs	Foodborne diseases
FDA	Food and Drug Administration
GABA	Gama-aminobutyric acid
GadD	Glutamate decarboxylase
GadT	GAD Transporters
GALT	Intestine-specific immune mechanism
*gbu*	Glycine betaine
HCl	Hydrochloric acid
HIV	Human Immunodeficiency Virus
Hsp60	LAP receptor
IKKα	IκB kinase
InlA, InlB, In1C and In1P	Internalins
LAMP	Loop-mediated isothermal amplification
LAP	Listeria adhesion protein
LIPI-1, LIPI-3, and LIPI-4	Pathogenicity islands
LLO	Cytolysin listeriolysin O
m	Maximum acceptable value per sample
M	Unacceptable value per sample.
MAPA	Ministry of Agriculture, Livestock and Supply
MLCK	Myosin light chain kinase
n	Number of sample units
NF-κB	Nuclear factor kappaB
NH_3_	Ammonia
PC-PLC	Broad-spectrum phospholipase C
PCR	Polymerase chain reaction
PI-PLC	Phosphatidylinositol-specific phospholipase C
POAO	Products derived from animals
PrfA	Transcriptional regulator
QACs	Quaternary ammonium compounds
QS	Quorum sensing
RDC	Collegiate board resolution
RTE	Ready-to-eat
RT-PCR	Real-time polymerase chain reaction
TSAYE	Tryptone soy yeast extract agar
